# Supplementary material for: The interactome of intact mitochondria by cross-linking mass spectrometry provides evidence for coexisting respiratory supercomplexes
Source: Mol Cell Proteomics. 2017 Dec 8;17(2):216–32. doi: 10.1074/mcp.RA117.000470 (PMC5795388; doi:10.1074/mcp.RA117.000470)
Supplement: Supplemental Data [file supp_RA117.000470_133922_0_supp_23978_fzffwf.zip › spectra_annotation/mito_spectra_annotation/412-1-8-1-8-1.pdf]

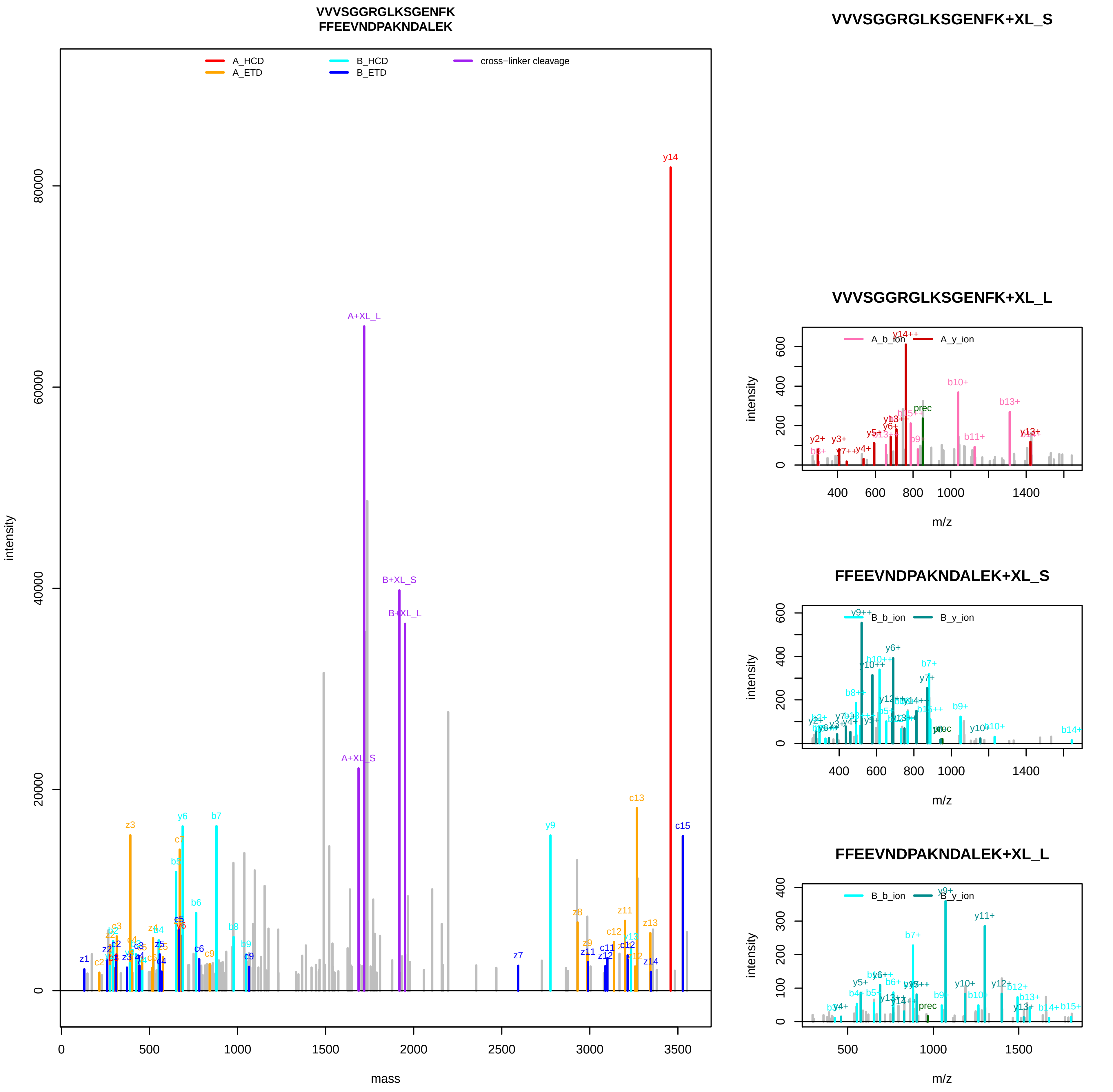

VVVSGGRGLKSGENFK
FFEEVNDPAKNDALEK
A_HCD
A_ETD
B_HCD
B_ETD
cross−linker cleavage
y14
A+XL_L
B+XL_S
B+XL_L
A+XL_S
c13
c15
y9
y6
b7
z3
c7
b6
z7
z14
intensity
mass
VVVSGGRGLKSGENFK+XL_S
VVVSGGRGLKSGENFK+XL_L
A_b_ion
A_y_ion
y14++
b10+
b13+
prec
b11+
y13+
FFEEVNDPAKNDALEK+XL_S
B_b_ion
B_y_ion
y9++
y6+
b7+
y7+
b9+
y10++
b14+
FFEEVNDPAKNDALEK+XL_L
y9+
y11+
b7+
y10+
y12+
m/z
